# Flavonoids from *Halostachys caspica* and Their Antimicrobial and Antioxidant Activities

**DOI:** 10.3390/molecules15117933

**Published:** 2010-11-05

**Authors:** Hao Liu, Yan Mou, Jianglin Zhao, Jihua Wang, Ligang Zhou, Mingan Wang, Daoquan Wang, Jianguo Han, Zhu Yu, Fuyu Yang

**Affiliations:** 1 College of Agronomy and Biotechnology, China Agricultural University, Beijing 100193, China; 2 College of Science, China Agricultural University, Beijing 100193, China; 3 College of Animal Science and Technology, China Agricultural University, Beijing 100193, China

**Keywords:** Chenopodiaceae, *Halostachys caspica*, flavonoids, antimicrobial activity, antioxidant activity

## Abstract

Seven flavonoids have been isolated from the aerial parts of *Halostachys caspica* C. A. Mey. (Chenopodiaceae) for the first time. By means of physicochemical and spectrometric analysis, they were identified as luteolin (**1**), chrysin (**2**), chrysin 7-*O*-β-D-glucopyranoside (**3**), quercetin (**4**), quercetin 3-*O*-β-D-glucopyranoside (**5**), isorhamentin-3-*O*-β-D-glucopyranoside (**6**), and isorhamentin-3-*O*-β-D-rutinoside (**7**). All flavonoids were evaluated to show a broad antimicrobial spectrum of activity on microorganisms including seven bacterial and one fungal species as well as pronounced antioxidant activity. Among them, the aglycones with relatively low polarity had stronger bioactivity than their glycosides. The results suggested that the isolated flavonoids could be used for future development of antimicrobial and antioxidant agents, and also provided additional data for supporting the use of *H. caspica* as forage.

## 1. Introduction

The plant kingdom, with its remarkable diversity of natural compounds, has merited special interest [1]. Among these compounds, flavonoids have received much research and development attention [2,3,4]. They not only function as stress protectants in plants (*i.e.,* pathogen defenders [5,6], and UV protectants [7,8]), but also have multi-beneficial biological activities such as antioxidative [9,10], anticarcinogenic [11], antimicrobial [12,13], antimutagenic [14], anti-inflammatory [15], antiallergic [16] and anti-obesity [17] properties.

*Halostachys caspica* C. A. Mey. belongs to the Chenopodiaceae family and is mainly distributed in the Provinces of Xinjiang and Gansu of Northwest China. It has been used in desert area as a high yield forage with good nutritional properties [18]. To the best of our knowledge, there are no reports on its phytochemical composition except for study of its lipids [19]. Our previous study showed that the ethyl acetate fraction of the crude ethanol extract of the aerial parts of *H. caspica* exhibited an obvious antimicrobial activity [20]. In this work, this ethyl acetate fraction of the crude ethanol extract was subjected to bioassay-guided fractionation leading to the isolation of seven flavonoids, including three different flavones and four different flavonols. The antimicrobial activities of these compounds were evaluated by testing their inhibitory ability on seven bacterial and one fungal species by using the dilution-colorimetric assay. In addition their antioxidant activity was tested by using two complementary systems, namely the DPPH free radical-scavenging and β-carotene-linoleic acid bleaching assays. Meanwhile, based on their bioactivities and structural characteristics, the structure-bioactivity relations of the compounds were also examined.

## 2. Results and Discussion

### 2.1. Elucidation of the purified flavonoids

**Figure 1:**
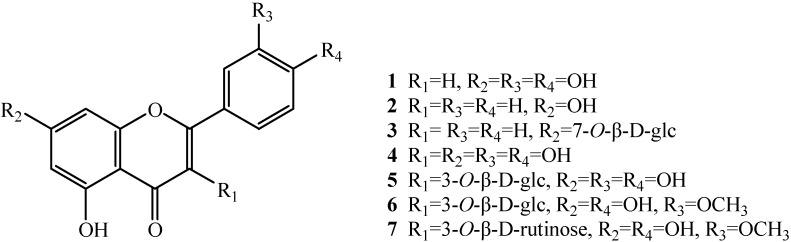
Chemical structures of compounds **1**-**7**.

Seven flavonoids were isolated from the ethyl acetate fraction of the crude ethanol extract of the aerial parts of *H. caspica* based on a bioassay-guided fractionation. After comparing their physicochemical and spectrometric data with those reported in literature, they were identified as known compounds and confirmed as luteolin (**1**) [21], chrysin (**2**) [22], chrysin 7-*O*-β-D-gluco-pyranoside (**3**) [23], quercetin (**4**) [24], quercetin 3-*O*-β-D-glucopyranoside (**5**) [25,26], isorhamentin-3-*O*-β-D-glucopyranoside (**6**) [25,27], and isorhamentin-3-*O*-β-D-rutinoside (**7**) [24], whose structures are shown in Figure 1.

There are about 100 genera including 1,400 species in Chenopodiaceae family around the World [18]. Some investigations on chenopodiaceous flavonoids have been reported [28,29,30,31,32,33,34,35,36,37]. Three flavonol aglycones (kaempferol, herbacetin and quercetin) and their three glycosides were isolated from the aerial parts of *Chenopodium murale* [28]. Three kaempferol glycosides [29], as well as seven flavonoids, including quercetin, kaempferol and five kaempferol glycosides were also isolated from *Chenopodium murale* [30]. Kaempferol, kaempferol 3-*O*-β-glucoside, kaempferol 3-*O*-β-diglucoside, kaempferol-3-*O*-arabinoglucoside, quercetin, quercetin 3-*O*-xylosylglucoside, and quercetin-3-*O*-rhamnoglucoside were isolated from the aerial parts of *Chenopodium album* [31,32]. Kaempferol 3-*O*-β-glucoside and quercetin 3-*O*-β-glucoside were isolated from the aerial parts of *Chenopodium opulifolium* [31]. Both quercetin-3-*O*-rhamnoglucoside and kaempferol-3-*O*-galactoside were isolated and identified from the leaves of *Chenopodium hircinum* [32]. Five flavonoids: isoquercitrin, isorhamnetin 3-*O*-β-D-glucopyranoside, isorhamnetin, rhamnazin, and 3,4',5-trihydroxy-3'-methoxy-flavone 7-*O*-β-D-glucosaminopyranoside were isolated from the epigeal parts of *Halocnemum strobilaceum* [33]. Two flavonoids: quercetin and quercetin 7-*O*-rhamnoside were isolated from *Hammada elegans* [34]. Four flavonoids: atriplexoside A, atriplexoside B, apigenin 7-*O*-β-D-glucoside, quercetin 3-*O*-β-L-rhamnoside were isolated from *Atriplex portulacoides* [35]. All the flavonoids from chenopodiaceous plants reported above could be classified into two groups: flavones and flavonols. The seven flavonoids isolated for the first time from *Halostachys caspica* in this study are also either flavones (*i.e.,* **1**, **2** and **3**) or flavonols (*i.e.,* **4**, **5**, **6**, and **7**). The chemical results in this study support the notion that *Halostachys* is one of the genera in Chenopodiaceae. These flavones and flavonols may have chemotaxonomic significance for chenopodiaceous plants, though more research is needed to confirm this.

### 2.2. Antimicrobial activity

All the flavonoids were tested for antimicrobial activities and the corresponding MIC and IC_50_ values are summarized in Table 1 and Table 2, respectively. Among them, chrysin (**2**) was the most active compound, with MIC values ranging from 6.25 μg/mL to 12.5 μg/mL on the test bacteria, and 50 μg/mL on the spore germination of *Magnaporthe oryzae*, respectively. For seven flavonoids, three aglycones such as luteolin (**1**), chrysin (**2**), and quercetin (**4**) were screened to show strong antimicrobial activity. The glycosides (*i.e.,* **3**, **5**, **6**, and **7**) exhibited relatively weak antimicrobial activity. The relations between structure and activity of seven flavonoids in this study seem to suggest that aglycones with relatively low polarity had stronger antimicrobial activity than their corresponding glycosides. Furthermore, by comparing luteolin (**1**) and chrysin (**2**), it can be speculated that the hydroxyl groups at the C-3' and C-4' positions were unfavorable to the antimicrobial activity for flavone derivatives. It is noteworthy that quercetin (**4**) had its most inhibitory activity on *Staphylococcus aureus*, with MIC and IC_50_ values of 6.25 μg/mL and 5.68 μg/mL, respectively.

Chrysin isolated from *Desmos chinensis* (Annonaceae) was also screened to exhibit a significant antibacterial activity against a number of Gram-positive and Gram-negative bacteria [36]. Luteolin from two moraceous plants, *Ficus chlamydocarpa* and *Ficus cordata*, was screened to have antimicrobial activity [37]. Quercetin from *Polygonum equisetiforme* (Polygonaceae) was screened to have antifungal activity on *Candida tropicalis* [38]. Both luteolin and quercetin were shown to have antibacterial activity on *Escherichia coli* [39].

**Table 1:** MIC values of the flavonoids from *H. caspica* on microorganisms.

Test Microorganism	MIC (μg/mL)							
	1	2	3	4	5	6	7	CK^+^
*A. tumefaciens*	200	12.5	400	100	200	200	400	20
*E. coli*	100	6.25	>400	100	>400	200	400	20
*P. lachrymans*	200	12.5	400	100	200	200	400	20
*X. vesicatoria*	100	6.25	200	100	>400	200	>400	20
*B. subtilis*	200	12.5	200	100	200	200	400	10
*S. aureus*	50	6.25	>400	6.25	100	400	400	100
*S. haemolyticus*	50	12.5	400	50	200	200	>400	20
*M. oryzae*	100	50	150	80	300	400	400	100

Note: The positive controls (CK^+^) on bacteria and *M. oryzae* were streptomycin sulfate and carbendazim, respectively.

**Table 2:** IC_50_ values of the flavonoids from *H. caspica* on microorganisms.

Test Microorganism	IC_50_ (μg/mL)							
	1	2	3	4	5	6	7	CK^+^
*A. tumefaciens*	92.96	6.48	293.67	63.23	99.72	92.17	202.30	8.34
*E. coli*	40.65	4.07	nd	60.37	nd	104.38	267.55	10.47
*P. lachrymans*	106.66	6.71	242.26	48.42	152.30	141.97	271.39	9.01
*X. vesicatoria*	44.74	5.81	163.53	63.01	nd	139.60	nd	11.62
*B. subtilis*	108.89	5.12	147.76	57.35	167.61	140.51	297.71	4.98
*S. aureus*	27.40	5.85	nd	5.68	56.00	248.03	194.57	78.60
*S. haemolyticus*	27.87	7.62	275.93	32.57	134.15	136.05	nd	7.75
*M. oryzae*	48.62	32.69	109.27	41.45	130.62	179.18	230.39	38.45

Note: The positive controls (CK^+^) on bacteria and *M. oryzae* were streptomycin sulfate and carbendazim, respectively. The 'nd' means not detected.

### 2.3. Antioxidant activity

Both DPPH radical scavenging and β-carotene-linoleic acid bleaching assays were used to test the antioxidant activities of the flavonoids (Table 3). The DPPH free radical scavenging assay determines the antiradical power of antioxidants [40]. Regarding the IC_50_ values, three aglycones (*i.e.,* **1**, **2** and **4**) showed strong antiradical activities. Quercetin (**4**) was the most active, with an IC_50_ value of 2.23 μg/mL, which was even better than that of BHT. All the glycosides showed relatively weak antiradical activities.

In β-carotene-linoleic acid assay, the antioxidant transfers hydrogen atoms to the peroxyl (R_1_R_2_CHOO^-^) radicals formed from the oxidation of linoleic acid and converts them to hydroperoxides (R_1_R_2_CHOOH) leaving β-carotene molecules intact [41]. The flavonoid compounds and compounds **6** and **7** had strong β-carotene quenching activity, with IC_50_ values ranging from 14.01 μg/mL to 140.48 μg/mL. The antioxidant results of flavonoids in this study also showed good consistency between the two complementary DPPH radical scavenging and β-carotene-linoleic acid bleaching assays, that indicated the antioxidant activity of aglycones was better than that of their glycosides.

Quercetin is a well-known antioxidant which has many beneficial health effects such as antiviral, anti-inflammatory, antibacterial, and muscle-relaxing properties. Furthermore, it improves normal cell survival and as a pro-oxidant it induces apoptosis in cancerous cells whereby it prevents tumor proliferation [42,43]. Luteolin and querctin isolated from fruits of *Euterpe oleracea* Mart. (Arecaceae) were screened to have strong antioxidant activity [44]. Otherwise, luteolin was approved to have anti-inflammatory, anti-allergic and anti-tumor activities [45,46,47]. There were also a few reports on antioxidant activity of chrysin [48,49].

**Table 3:** Antioxidant activity of the flavonoids from *H. caspica*.

Assay	IC_50_ (μg/mL)							
	1	2	3	4	5	6	7	BHT
DPPH inhibition	25.75	36.67	102.35	2.23	82.55	165.62	177.91	18.80
β-Carotene bleaching	67.66	38.23	140.48	14.01	97.52	393.37	210.62	31.46

## 3. Experimental

### 3.1. General

Silica gel (100-200 and 200-300 mesh, Qingdao Marine Chemical Company, China), Sephadex LH-20 (Pharmacia), and C_18_ reversed-phase silica gel (YMC) were used for column chromatography (CC). Thin layer chromatography (TLC) plates (Qingdao Marine Chemical Company, China) were coated with 0.5-mm and 1-mm layers of silica gel (GF_254_, 300-400 mesh), respectively. Melting points were determined on an XT4-100B microscopic melting-point apparatus (Tianjin Tianguang Optical Instruments Company, China) and are uncorrected. NMR spectra were recorded on a Bruker-ARX-300 (^1^H at 300 MHz and ^13^C at 75 MHz) or a Bruker Avance DRX-500 (^1^H at 500 MHz and ^13^C at 125 MHz) spectrometer. ESI-MS spectra were recorded on a Bruker Esquire 6000 LC/MS spectrometer. EI-MS spectra were registered on a Thermo Finnigan LCQ mass spectrometer. A microplate spectrophotometer (PowerWave HT, BioTek Instruments, USA) was employed to measure the light absorption value. β-Carotene, carbendazim, streptomycin sulfate, and 1,1-diphenyl-2-picrylhydrazyl (DPPH) were purchased from Sigma-Aldrich (USA). Linoleic acid was obtained from Johnson Matthey (UK). 3-(4,5-Dimethylthiazol-2-yl)-2,5-diphenyl tetrazolium bromide (MTT) was purchased from Amresco (USA). Butylated hydroxytoluene (BHT) and Tween-40 were from Beijing Chemical Company. All other chemicals and reagents were of analytical grade.

### 3.2. Plant material

The aerial parts of *Halostachys caspica* C. A. Mey. were collected in August 2007 at Shihezi of Xinjiang Province of China, and was authenticated by Professor Pin Yan of Shihezi University of Xinjiang. A voucher specimen of this collection (BSMPMI-200708001) was deposited at the Herbarium of the Institute of Chinese Medicinal Materials, China Agricultural University. The plant materials were left to dry in the shade at room temperature to a constant weight.

### 3.3. Extraction, fractionation and identification of the flavonoids

The air-dried and powdered aerial parts (7.23 kg) of *H. caspica* were soaked three times in 95% ethanol (30 L) at room temperature for an interval of 10 days. After the combined filtrates were concentrated under vacuum at 50 ºC, the brown residue (1,640 g) was suspended in water. It was extracted with petroleum ether, then with EtOAc, and last with *n*-BuOH. The EtOAc extract (41.96 g) was subjected to silica gel column chromatography with a gradient of CHCl_3_-MeOH (from 1:0 to 1:1, v/v) as an eluent, and six fractions (A, B, C, D, E and F) were collected based on TLC analysis. Fraction B (0.42 g) was subjected to silica gel column chromatography with a gradient of petroleum ether-acetone (from 1:0 to 0:1, v/v) as an eluent, and four sub-fractions were collected. Sub-fraction B-1 (0.06 g) obtained by elution with petroleum ether-acetone (3.5:1.0, v/v) was further purified over Sephadex LH-20 to afford **1** (9 mg) and **2** (12 mg). Sub-fraction B-2 (0.10 g) obtained with the eluent petroleum ether-acetone (1:1, v/v) was further purified on Sephadex LH-20 and reverse phase chromatography (RP-18) to afford **4** (10 mg), **5** (20 mg), and **6** (19 mg). Fraction E (0.30 g) was re-subjected to silica gel column chromatography with a gradient of CHCl_3_-MeOH (from 1:0 to 1:1, v/v) as an eluent to afford **3** (15 mg) and **7** (7 mg). The physicochemical and spectrometric data of seven flavonoids were as follows:

*Luteolin* (**1**). Yellow needles (MeOH); m.p. 328-330 ºC; ESI-MS *m*/*z* 287 [M+H]^+^; ^1^H-NMR (DMSO-*d*_6_, 500 MHz) δ (ppm), 6.76 (1H, s, H-3), 12.93 (1H, s, OH-5), 6.29 (1H, d, *J* = 2.0 Hz, H-6), 9.52 (1H, s, OH-7), 6.47 (1H, d, *J* = 2.0 Hz, H-8), 7.75 (1H, d, *J* = 2.2 Hz, H-2'), 6.90 (1H, d, *J* = 8.5 Hz, H-5'), 7.61 (1H, dd, *J* = 2.2, 8.5 Hz, H-6'); ^13^C-NMR (DMSO-*d*_6_, 125 MHz) δ (ppm), 167.6 (C-2), 101.8 (C-3), 181.0 (C-4), 161.3 (C-5), 99.6 (C-6), 163.6 (C-7), 94.3 (C-8), 157.5 (C-9), 102.3 (C-10), 119.8 (C-1'), 112.2 (C-2'), 146.4 (C-3'), 152.0 (C-4'), 115.8 (C-5'), 118.9 (C-6'). The structure was confirmed by comparison with literature data [21].

*Chrysin* (**2**). Yellow powder (MeOH); m.p. 280-282 ºC; ESI-MS *m*/*z* 255 [M+H]^+^, 253 [M-H]^-^; ^1^H-NMR (DMSO-*d*_6_, 500 MHz) δ (ppm ), 6.96 (1H, s, H-3), 12.81 (1H, s, OH-5), 6.21 (1H, d, *J* = 2.0 Hz, H-6), 10.91 (1H, s, OH-7), 6.52 (1H, d, *J* = 2.0 Hz, H-8), 8.06 (2H, br d, *J* = 8.0 Hz, H-2', 6'), 7.59 (3H, m, H-3', 4', 5'); ^13^C-NMR (DMSO-*d*_6_, 125 MHz) δ (ppm), 163.1 (C-2), 105.2 (C-3), 181.8 (C-4), 161.4 (C-5), 99.0 (C-6), 164.4 (C-7), 94.1 (C-8), 157.4 (C-9), 104.0 (C-10), 130.7 (C-1'), 126.4 (C-2', 6'), 129.1 (C-3', 5'), 132.0 (C-4'). The structure was confirmed by comparison with literature data [22].

*Chrysin 7-O-β-D-glucoside* (**3**). Yellow amorphous powder (MeOH); m.p. 218-221 ºC; ESI-MS *m*/*z* 417 [M+H]^+^; ^1^H-NMR (DMSO-*d*_6_, 500 MHz) δ (ppm), 6.95 (1H, s, H-3), 12.81 (1H, s, OH-5), 6.39 (1H, br s, H-6), 6.82 (1H, br s, H-8), 8.09 (2H, br d, *J* = 7.0 Hz, H-2', 6'), 7.59 (3H, m, H-3', 4', 5'), 5.12 (1H, d, *J* = 7.0 Hz, glc-H-1), 5.44 (1H, d, *J* = 4.6 Hz, glc-OH-2), 5.17 (1H, d, *J* = 4.2 Hz, glc-OH-3), 5.10 (1H, d, *J* = 5.0 Hz, glc-OH-4), 4.64 (1H, t, *J* = 5.5 Hz, glc-OH-6), 3.19-3.70 (m, sugar protons); ^13^C-NMR (DMSO-*d*_6_, 125 MHz) δ (ppm), 163.6 (C-2), 105.4 (C-3), 182.0 (C-4), 161.3 (C-5), 99.5 (C-6), 163.9 (C-7), 94.7 (C-8), 157.3 (C-9), 105.2 (C-10), 130.7 (C-1'), 126.5 (C-2'), 129.1 (C-3'), 132.1 (C-4'), 129.2 (C-5'), 126.3 (C-6'), 99.7 (glc-C-1), 72.9 (glc-C-2), 76.6 (glc-C-3), 69.2 (glc-C-4), 77.4 (glc-C-5), 60.7 (glc-C-6). The structure was confirmed by comparison with literature data [23].

*Quercetin* (**4**). Yellow amorphous powder (MeOH); m.p. 314-315 ºC; ESI-MS *m*/*z* 303 [M+H]^+^, 301 [M-H]^-^; ^1^H-NMR (DMSO-*d*_6_, 500 MHz) δ (ppm), 9.56 (1H, s, OH-3), 12.48 (1H, s, OH-5), 6.17 (1H, d, *J* = 2.0 Hz, H-6), 10.76 (1H, s, OH-7), 6.39 (1H, d, *J* = 2.0 Hz, H-8), 7.66 (1H, d, *J* = 2.0 Hz, H-2'), 9.33 (1H, s, OH-3'), 9.33 (1H, s, OH-4'), 6.87 (1H, d, *J* = 8.5 Hz, H-5'), 7.53 (1H, dd, *J*=2.0, 8.0 Hz, H-6'); ^13^C-NMR (DMSO-*d*_6_, 125 MHz) δ (ppm), 147.9 (C-2), 135.9 (C-3), 176.0 (C-4), 160.9 (C-5), 98.4 (C-6), 164.1 (C-7), 93.5 (C-8), 156.3 (C-9), 103.2 (C-10), 122.1 (C-1'), 115.2 (C-2'), 145.2 (C-3'), 147.0 (C-4'), 115.8 (C-5'), 120.2 (C-6'). The structure was confirmed by comparison with literature data [24].

*Quercetin 3-O-β-D-glupyranoside* (**5**). Yellow amorphous powder (MeOH); m.p. 224-226 ºC; EI-MS *m/z* 463 [M-H]^-^; ^1^H-NMR (DMSO-*d*_6_, 500 MHz) δ (ppm), 12.60 (1H, s, OH-5), 6.21 (1H, d, *J* = 2.0 Hz, H-6), 10.80 (1H, s, OH-7), 6.41 (1H, d, *J* = 2.0 Hz, H-8), 7.61 (1H, d, *J* = 2.2 Hz, H-2'), 9.65 (1H, s, OH-3'), 9.14 (1H, s, OH-4'), 6.86 (1H, d, *J* = 9.0 Hz, H-5'), 7.58 (1H, dd, *J* = 2.2, 9.0 Hz, H-6'), 5.47 (1H, d, *J* = 7.4 Hz, glc-H-1), 3.10-3.60 (m, sugar protons); ^13^C-NMR (DMSO-*d*_6_, 125 MHz) δ (ppm), 159.1 (C-2), 136.2 (C-3), 179.6 (C-4), 162.2 (C-5), 98.6 (C-6), 165.6 (C-7), 94.4 (C-8), 159.1 (C-9), 104.6 (C-10), 122.0 (C-1'), 116.0 (C-2'), 145.0 (C-3') , 150.2 (C-4'), 116.9 (C-5'), 122.7 (C-6'), 100.6 (glc-C-1), 74.9 (glc-C-2), 77.9 (glc-C-3), 70.2 (glc-C-4), 76.0 (glc-C-5), 61.7 (glc-C-6). The structure was confirmed by comparison with literature data [25,26].

*Isorhamnetin 3-O-β-D-glucopyranoside* (6). Yellow amorphous powder (MeOH); m.p. 267-269 ºC; ESI-MS *m*/*z* 479 [M+H]^+^; ^1^H-NMR (MeOD, 500 MHz) δ (ppm), 6.20 (1H, d, *J* = 2.0 Hz, H-6), 6.39 (1H, d, *J* = 1.8 Hz, H-8), 7.57 (1H, d, *J* = 2.0 Hz, H-2'), 3.90 (3H, s, OCH_3_-3'), 7.05 (1H, d, *J* = 8.4 Hz, H-5'), 7.69 (1H, dd, *J* = 1.8, 8.4 Hz, H-6'), 5.40 (1H, d, *J* = 8.5 Hz, glc-H-1), 3.07-3.83 (m, sugar protons); ^13^C-NMR (MeOH, 125 MHz) δ (ppm), 156.2 (C-2), 133.0 (C-3), 177.5 (C-4), 161.1 (C-5), 98.8 (C-6), 164.0 (C-7), 93.8 (C-8), 156.4 (C-9), 104.2 (C-10), 121.7 (C-1'), 111.5 (C-2'), 149.9 (C-3'), 146.4 (C-4'), 115.6 (C-5'), 122.3 (C-6'), 55.7 (OCH_3_-3'), 101.0 (glc-C-1), 74.2 (glc-C-2), 77.2 (glc-C-3), 70.0 (glc-C-4), 76.6 (glc-C-5), 61.2 (glc-C-6). The structure was confirmed by comparison with literature data [25,27].

*Isorhamentin 3-O-β-D-rutinoside* (**7**). Yellow amorphous powder (MeOH); m.p. 186-187 ºC; ESI-MS *m*/*z* 623 [M-H]^-^, 647 [M+Na]^+^; ^1^H-NMR (DMSO-*d*_6_, 300 MHz) δ (ppm), 12.50 (1H, s, OH-5), 6.19 (1H, d, *J* = 1.9 Hz, H-6), 10.89 (1H, s, OH-7), 6.41 (1H, d, *J* = 1.8 Hz, H-8), 7.86 (1H, d, *J* = 2.0 Hz, H-2'), 3.83 (3H, s, OCH_3_-3'), 9.83 (1H, s, OH-4'), 6.92 (1H, d, *J* = 8.4 Hz, H-5'), 7.53 (1H, dd, *J* = 2.0, 8.4 Hz, H-6'), 5.44 (1H, d, *J* = 7.3 Hz, glc-H-1), 4.41 (1H, d, *J* = 10.8 Hz, rha-H-1), 0.98 (1H, d, *J* = 5.6 Hz, rha-H-6), 3.07-3.83 (m, sugar protons); ^13^C-NMR (DMSO-*d*_6_, 75 MHz) δ (ppm), 156.7 (C-2), 133.2 (C-3), 177.4 (C-4), 161.4 (C-5), 99.1 (C-6), 165.0 (C-7), 94.1 (C-8), 156.5 (C-9), 103.9 (C-10), 121.2 (C-1'), 113.5 (C-2'), 149.6 (C-3'), 55.9 (OCH_3_-3'), 147.1 (C-4'), 115.5 (C-5'), 122.5 (C-6'), 101.5 (glc-C-1), 74.5 (glc-C-2), 76.6 (glc-C-3), 70.8 (glc-C-4), 76.1 (glc-C-5), 67.0 (glc-C-6), 101.1 (rha-C-1), 70.5 (rha-C-2), 70.8 (rha-C-3), 72.0 (rha-C-4), 68.5 (rha-C-5), 17.8 (rha-C-6). The structure was confirmed by comparison with literature data [24].

### 3.4. Antimicrobial activity

#### 3.4.1. Antibacterial activity assay

Four Gram-negative (*Agrobacterium tumefaciens* ATCC 11158, *Escherichia coli* ATCC 29425, *Pseudomonas lachrymans* ATCC 11921 and *Xanthomonas vesicatoria* ATCC 11633) and three Gram-positive (*Bacillus subtilis* ATCC 11562, *Staphylococcus aureus* ATCC 6538 and *Staphylococcus haemolyticus* ATCC 29970) bacteria were selected for the antibacterial activity assay. They were grown in liquid LB medium (yeast extract 5 g/L, peptone 10 g/L, NaCl 5 g/L, pH 7.0) overnight at 28 ºC, and the diluted bacterial suspension (10^6^ cfu/mL) was ready for detection. A modified broth dilution-colorimetric assay by using the chromogenic reagent 3-(4,5-dimethylthiazol-2-yl)-2,5-diphenyl tetrazolium bromide (MTT) was used to detect the antibacterial activity of the flavonoids according to our previous report [50]. Briefly, the sample was dissolved in acetone at an initial concentration of 10 mg/mL. Then it was diluted with 30% acetone to obtain concentrations ranging from 0.25 mg/mL to 3.0 mg/mL. Test sample solutions (10 μL) and prepared bacterial suspension (90 μL) containing 1 × 10^6^ cfu/mL were added into each well of the 96-well microplate. The negative control well contained 90 μL of the inoculum (1 × 10^6^ cfu/mL) and 10 μL of 30% acetone. Streptomycin sulfate was used as the positive control. After the plates were agitated to mix the contents of the wells using a plate shaker and incubated in the dark at 28 ºC for 24 h, 10 μL of MTT (5 mg/mL in 0.2 mol/L, pH 7.2 phosphate-buffered saline) was added into each well, and the plates were incubated for another 4 h. The minimum inhibitory concentration (MIC) value was defined as the lowest sample concentration that inhibited visible growth, as indicated by the MTT staining. Only living microorganisms could convert MTT to formazan and a blue color appeared in the well [51].

To further determine the median inhibitory concentration (IC_50_) value of the samples, the above microplates incubated with MTT were centrifuged at 1,500 *g* for another 20 min. Then the supernatant was aspirated, 150 μL of dimethyl sulfoxide (DMSO) was added into each well, and the colored formazan products were extracted for 30 min. After complete extraction, the plate was centrifuged at 1,500 g for another 20 min, and then 100 μL of the supernatant (DMSO solution) in each well was transferred to a corresponding well of another 96-well microplate to measure their light absorption values at wavelength 510 nm using a microplate spectrophotometer. The percentage (%) of the bacterial growth inhibition was determined as [(*A*_c_–*A*_t_)/*A*_c_] × 100, where *A*_c_ was an average of six replicates of light absorption values at wavelength 510 nm of the negative controls, and *A*_t_ was an average of six replicates of light absorption values at wavelength 510 nm of the samples. The IC_50_ value was calculated using the linear relation between the inhibitory probability and concentration logarithm according to the method of Sakuma [52].

#### 3.4.2. Antifungal activity assay

Rice blast fungus, *Magnaporthe oryzae* (strain P131) was maintained on the oatmeal-tomato agar medium (oatmeal 30 g/L, tomato juice 150 mL/L, and agar 20 g/L) at 25 ºC. The spores were prepared from 7-day-old cultures of *M. oryzae*, according to our previous reports [50,53]. The sample-acetone solution (25 μL) was mixed with an equivalent volume of spore suspension containing 2 × 10^6^ spores per mL. The mixture was then placed on separate concave glass slides. The final concentrations of the samples ranged from 0.10 to 0.35 mg/mL containing 5% (v/v) acetone. The negative control was 5% acetone, and the positive control was carbendazim at concentrations ranging from 0.01 to 0.10 mg/mL. Three replicates were used for each treatment. Slides containing the spores were incubated in a moist chamber at 25 ºC for 7 h. Each slide was then observed under the microscope for spore germination status. About 100 spores per replicate were observed to detect spore germination. The percentage (%) of spore germination inhibition was determined as [(*G*_c_–*G*_t_)/*G*_c_] × 100, where *G*_c_ is an average of three replicates of germinated spore numbers in the negative control, and *G*_t_ is an average of three replicates of germinated numbers in the treated sets. The IC_50_ value calculation for the spore germination inhibition was the same as that for antibacterial activity assay. The MIC value on the spore germination was defined as the lowest sample concentration that inhibited visible spore germination.

### 3.5. Antioxidant activity

#### 3.5.1. DPPH radical scavenging assay

Radical scavenging assay was determined by a microplate spectrophotometric method based on the reduction of DPPH according to our previous report [54]. Briefly, DPPH solution (80 μL, 0.2 mg/mL) and flavonoid solution in 30% ethanol (20 μL) were added to each well of the microplate and mixed. The mixture was shaken vigorously and left to stand at 37 ºC for 30 min in the dark. The absorbance of the solution was then measured at wavelength 515 nm using a microplate spectrophotometer. Inhibition (%) of free radical (DPPH) in percent was determined as [(*A*_control_-*A*_sample_)/*A*_control_] × 100, where *A*_control_ is the absorbance of the control reaction containing all reagents except the test sample, and *A*_sample_ is the absorbance of the test flavonoid. Tests were carried out in triplicate. BHT was used as the positive control. The IC_50_ value was calculated using linear relation between the flavonoid concentration and probability of the percentage of DPPH inhibition.

#### 3.5.2. β-Carotene-linoleic acid bleaching assay

The antioxidant activity of the flavonoids was evaluated by the β-carotene-linoleic acid bleaching method according to our previous report [54]. Briefly, linoleic acid (25 μL) and Tween-40 (200 mg) were added in the β-carotene solution (0.5 mg of β-carotene dissolved in 1 mL of chloroform). Chloroform was then removed using a rotary evaporator at 50 ºC. Distilled water (50 mL) saturated with oxygen for 30 min at a flow rate of 100 mL/min were added and the mixture was vigorously shaken. The above β-carotene-linoleic acid-Tween mixture (90 μL) and the flavonoid solution (10 μL, concentrations from 0.05 mg/mL to 4.0 mg/mL) in 30% ethanol solution were added into each well. An equal amount of 30% ethanol was used as the control. The microplates were then placed in an incubator at 50 ºC for 2 h together with BHT as the positive control. The absorbance of the solution was then measured at wavelength 460 nm using a microplate spectrophotometer. The percentage (%) of β-carotene bleaching inhibition of each sample was determined as (*A*_β-carotene after 2 h assay_/*A*_initial β-carotene_) × 100, Where *A*_β-carotene after 2 h assay_ is the absorbance of the sample with β-carotene-linoleic acid mixture after 2 h period of incubation, and *A*_initial β-carotene_ is the absorbance of the initial mixture. All tests were carried out in triplicate. The IC_50_ value calculation for β-carotene bleaching inhibition was the same as that for antibacterial activity assay.

## 4. Conclusions

In this study, we reported for the first time that seven flavonoids from *H. caspica* showed a broad spectrum of antimicrobial activity on microorganisms including bacterial (Gram-positive and negative) and fungal species, as well as pronounced antioxidant activity. The bioactivities of flavonoid aglycones with relatively low polarity were better than those of the glycosides. The isolated flavonoids may be contributed to the antimicrobial and antioxidant activity of the extract and fraction, though some synergistic or antagonistic effects should be occurred in the either extract or fraction which need to be clarified. The present study suggested that the crude extract and fraction as well as the flavonoids from *H. caspica* could be used for future development of naturally-occurring antimicrobials and antioxidants. It also provided additional data for supporting the utilization of *H. caspica* as forage. A greater research effort should be devoted to confirm antimicrobial and antioxidant activities of the flavonoids from *H. caspica* which may be applied in food, agriculture and medicinal industry as a source of antimicrobial and antioxidative agents. The underlying antimicrobial and antioxidant mechanisms of the flavonoids, their contents in plant materials as well as their preparation in large scale also need to be further studied.
